# Coffee Silverskin: Chemical and Biological Risk Assessment and Health Profile for Its Potential Use in Functional Foods

**DOI:** 10.3390/foods11182834

**Published:** 2022-09-13

**Authors:** Agata Nolasco, Jonathan Squillante, Francesco Esposito, Salvatore Velotto, Raffaele Romano, Maria Aponte, Antonella Giarra, Maria Toscanesi, Emma Montella, Teresa Cirillo

**Affiliations:** 1 Department of Agricultural Sciences, University of Naples Federico II, Via Università, Portici, 100-80055 Naples, Italy; 2 Department of Public Health, University of Naples Federico II, Via Sergio Pansini, 5-80131 Naples, Italy; 3 Department of Promotion of Human Sciences and the Quality of Life, University of Study of Roma “San Raffaele”, Via di Val Cannuta, 247-00166 Roma, Italy; 4 Department of Chemical Sciences, University of Naples “Federico II”, Via Cintia, 21-80126 Naples, Italy

**Keywords:** silverskin, coffee by-products, risk assessment, agri-food waste, dietary exposure

## Abstract

The coffee supply chain is characterized by a complex network with many critical and unsustainable points producing a huge amount of waste products. Among these, coffee silverskin (CS), the only by-product of the coffee roasting phase, has an interesting chemical profile that suggests potential use as a food ingredient. However, few data on its safety are available. For this reason, the purpose of the study was to assess the occurrence of chemical and biological contaminants in CS, and the resulting risk due to its potential consumption. Essential, toxic, and rare earth elements, polycyclic aromatic hydrocarbons (PAHs), process contaminants, ochratoxin A (OTA), and pesticides residues were analyzed in three classes of samples (*Coffea arabica* CS, *Coffea robusta* CS, and their blend). Furthermore, total mesophilic bacteria count (TMBC) at 30 °C, Enterobacteriaceae, yeasts, and molds was evaluated. The risk assessment was based upon the hazard index (HI) and lifetime cancer risk (LTCR). In all varieties and blends, rare earth elements, pesticides, process contaminants, OTA, and PAHs were not detected except for chrysene, phenanthrene, and fluoranthene, which were reported at low concentrations only in the *arabica* CS sample. Among essential and toxic elements, As was usually the most representative in all samples. Microorganisms reported a low load, although *arabica* and *robusta* CS showed lower contamination than mixed CS. Instead, the risk assessment based on the potential consumption of CS as a food ingredient did not show either non-carcinogenic or carcinogenic risk. Overall, this study provides adequate evidence to support the safety of this by-product for its potential use in functional foods.

## 1. Introduction

Coffee is one of the most consumed beverages in the world and is the second most traded product, after petroleum, with a market volume of about $15 billion [1]. The primary coffee production comes from South America, particularly Brazil, which has recorded an 18.5% increase in production in 2019–2020 [2]. *Coffea arabica* is the most dominant variety in the coffee market, with 175,347 thousand 60 kg bags in 2020, compared to 70,086 thousand 60 kg bags for *Coffea canephora* var. *robusta* [2]. Due to the enormous amount of coffee produced, marketed, and consumed, the coffee supply chain faces an environmental pollution burden, also related to the disposal of by-products.

Coffee cherry undergoes numerous processing steps that transform the raw fruit into liquid coffee. These processing steps are made to separate the outer layers that cover the green coffee bean. The structure of the coffee bean is composed as follows: husk (exocarp), pulp (mesocarp), parchment (endocarp), silverskin (integument), and finally, the two beans that constitute the final product of the processing chain from which the beverage is obtained [3]. Briefly, reviewing the processing steps, the coffee cherry, after being harvested, with a first discharge of the unsuitable cherries, is subjected to a wet or dry process [4]. The wet method involves removing the pulp from the bean by fermentation.

On the other hand, the dry method involves drying and hulling the coffee cherry and collecting husk, pulp, and parchment, which are disposed of as waste [3]. At this point, the green coffee beans, obtained from the previous processing steps, are packaged and shipped to consumer countries where roasting occurs. The roasting phase is a crucial part of coffee processing; it influences the organoleptic characteristics of the final beverage [5]. The coffee roasting phase includes blending different coffee varieties, with a roasting temperature of about 200 °C for less than 20 min, with substantial variability in time and temperature depending on the roasting system adopted [6].

The only by-product of this step is the coffee silverskin (CS), a thin integument obtained by detachment from the green bean due to the high temperatures reached during roasting. Finally, the roasted coffee bean is ready to be used to obtain the well-known beverage. The CS has a low mass (1% to 2% of the whole bean’s weight), but the total amount of this by-product is considerable in relation to the coffee consumed each year. Some studies on its potential reuse in cosmetics, nutraceuticals, and industry [7,8,9]. Moreover, given its chemical-physical profile related to the good amount of protein (19%), dietary fiber (30–70%), phenolic compounds, and melanoidins, CS is earning much interest for possible use as food or food ingredient [10,11,12,13,14,15].

To be included in the list of novel foods, it must undergo the authorization procedure set by Regulation (EU) No. 2015/2283 [16]. At present, coffee silverskin is evaluated as a novel food requiring pre-market approval [17]. The European Commission is responsible for authorizing novel foods and, as part of the procedure, may ask the European Food Safety Authority (EFSA) to conduct a scientific risk assessment to establish their safety [18]. In order to use it as a novel food, it is necessary to evaluate the absence of chemical and microbiological contaminants in this product, thus proceeding to a risk assessment. To this end, the present study aims to provide a comprehensive characterization investigating the occurrence of heavy metals, pesticides, rare earth elements (REEs), polycyclic aromatic hydrocarbons (PAHs), and biological contaminants. Since chemical features of coffee could differ among varieties [19], the silverskin obtained from the roasting of *Coffea arabica*, *Coffea robusta*, and a blend of both were studied. Ultimately, a risk assessment based on deterministic simulation was carried out considering the potential consumption of CS as a food ingredient.

## 2. Materials and Methods

### 2.1. Coffee Silverskin Sample

The arabica and *robusta* silverskin were obtained by roasting *Coffea arabica* and *Coffea robusta* green beans. The roasting was assessed in the laboratory using a Probatino rotary drum roaster (Probat, Emmerich am Rhein, Germany), equipped with a display to monitor time and temperature. The controlled curves of roasting temperatures were set out according to [6]. The mixed silverskin was provided by two coffee industries of the Campania region (Italy), obtained by blending roasted green beans of *Coffea arabica* and *Coffea robusta* (in unspecified ratio). Coffee varieties are usually blended by the companies during the roasting process, then the CS is recovered with suction cyclones and collected [20]. All the CS samples were stored in the dark at 20 °C and analyzed within three days.

### 2.2. Chemical Analysis

#### 2.2.1. Essential, Toxic, and Rare Earth Elements

All solution preparation and sample dilution for multi-elemental analysis were made with high-purity water (resistivity of 18.2 MΩ cm) obtained from a Milli-Q unit (Millipore, Burlington, MA, USA). Nitric acid (HNO_3_, 69% *v*/*v* Ultratrace^®^ ppb-trace analysis grade) and hydrofluoric acid (HF, 48% *v*/*v* Ultratrace^®^ ppb-trace analysis grade) were provided by Scharlau (Barcelona, Spain). Multi-component certified solution of 30 elements (ultrapure grade for ICP) was provided by Ultrascientific (Bologna, Italy). Boric acid (99.97% trace metals basis) and multi-component certified solution of 16 rare earth elements REE (50 mg/L each, ultrapure grade for ICP, TraceCERT^®^) were purchased from Merck (Darmstadt, Germany). All glassware and plastic containers used for the preparation of samples and standards were tested and found free of analyzed elements.

Multi-elemental analysis was carried out by the Inductively Coupled Plasma—Mass Spectrometry (ICP-MS) and Microwave Plasma-Atomic Emission Spectrometry (MP-AES) after digestion.

An aliquot of 250 ± 1 mg of each sample was digested with 10 mL of ultrapure nitric acid (HNO_3_, 69% *v*/*v* Ultratrace^®^ ppb-trace analysis grade) and 1 mL of hydrofluoric acid (HF, 48% *v*/*v* Ultratrace^®^ ppb-trace analysis grade) in PP test tubes. The latter were placed in a water bath, preheated to 80 °C and proceeded to digestion for 2 h. After this time, 400 mg of boric acid were added to the mixture, and digestion continued for 1 h. Samples were brought to a final volume of 20 mL with HNO_3_ solution (2%, *v*/*v*) for the subsequent elemental analysis.

In this case, 19 elements (As, Sb, Ba, Be, B, Cd, Co, Cr, Fe, Mn, Hg, Ni, Pb, Cu, Se, Sn, Tl, V, Zn) and 15 REEs (Y, La, Ce, Pr, Nd, Sm, Eu, Gd, Tb, Dy, Ho, Er, Tm, Yb, Lu) were analyzed by Aurora M90 ICP-MS instruments (Bruker, Bremen, Germany). Na, K, Mg and Ca were analyzed by 4210 MP-AES instruments (Agilent, Santa Clara, CA, USA). Calibration curves were obtained in the range from 1 to 100 µg/L for ICP-MS and in the range from 0.1 to 100 mg/L for MP-AES for each analyzed element from certified standard solutions. A blank for reagents and a blank for the digestion method were performed to verify contamination of all materials, and calibration was verified in the instrumental sequence with two control standards for every 20 samples.

The limit of quantification (LOQ) was calculated by the method of blanks variability for each investigated element, and they are included in the range 0.05–1 mg/kg in the final sample for ICP-MS analysis and 10 mg/Kg in the final sample for metals detected with MP-AES.

#### 2.2.2. Polycyclic Aromatic Hydrocarbons (PAHs)

Sample preparation and solutions for PAHs analysis were made with acetone (≥ 99.8%, GC grade) and n-hexane (≥95%, GC grade). Multi-component certified solution of 15 PAHs (2000 µg/mL each) and deuterated internal standard mixture solution (100 µg/mL each) were provided by Ultrascientific (Bologna, Italy). All glassware was tested and found free of PAHs.

An aliquot of 1.000 g ± 1 mg of each sample was extracted with 25 mL acetone/n-hexane 1:1 *v*/*v* using an ultrasonic bath (Branson Ultrasonic Corporation, Brookfield, CT, USA) for three 30-min cycles in closed glass vials. After this time, the extract was concentrated to a final volume of 1 mL using automatic concentrator MultiVap 8 (Labtech, Bergamo, Italy), and the purification procedure was unnecessary for the subsequent GC-MS analysis. The extract was injected into a gas chromatograph coupled with a mass spectrometer (GC 2010 Plus, MS-TQ8030, Shimadzu, Kyoto, Japan) for the determination of 15 PAHs [21].

Calibration curves were obtained in the range from 1 to 100 µg/L by calculating the area ratio of the target ion to the respective internal standard with five standard solutions obtained from certified standard solutions. A blank for reagents and a blank for the digestion method were performed to verify contamination of all materials, and calibration was verified in the instrumental sequence with a control standard for every ten samples.

The limit of quantification (LOQ) was calculated by the method of blanks variability and was equal to 0.05 mg/kg in the final sample.

#### 2.2.3. Acrylamide (AA) and Furan

The acrylamide (AA) was analyzed following the [22] method with some modifications. A solid-phase extraction dispersed with C18 sorbent packed in polypropylene columns was performed by taking an aliquot of 0.5 g of homogenized sample. Then, the aqueous eluate was derivatized after bromination and subsequent GC-MS reading and quantification of the derivative. The limit of quantification (LOQ) was 0.02 mg/kg.

The furan was analyzed with headspace technique following the Park et al., 2021 [23] method. An aliquot of 5 g of sample and 5 mL of HPLC water were added to a headspace vial (20-mL) and carefully sealed. Then d4-furan solution (internal standard) was added to the vial, homogenized and placed on ice. Furan was extracted from the samples by an automated solid-phase micro-extraction (SPME) agitating the sample at 300 rpm, 50 °C. The fiber (carboxen/polydimethylsiloxane) was exposed to the headspace of vial samples for 20 min at 25 mm depth. Then, the fiber was thermally desorbed in the injection port of a gas chromatograph at 40 mm depth for 5 min. The limit of quantification (LOQ) was 0.1 mg/kg.

#### 2.2.4. Ochratoxin A (OTA)

The analysis of ochratoxin A (OTA) was performed following the official method EN 1413:2003 with some modifications. 10 g of sample were homogenized with methanol:water solution (70:30 *v*/*v*), and it was diluted with a phosphate buffer solution prepared by dissolving 160 g of NaCl, 4.0 g of KCl, and 36.0 g of sodium hydrogen phosphate dihydrate (Na_2_HPO_4_-2H_2_O) in 1800 mL of water, adjusting the pH of the solution to 7.4 with 0.1 M HCl or with 0.1 M NaOH. Then, purification was performed by immunoaffinity chromatography onto columns containing a gel on which anti-OTA antibodies are immobilized. After, mycotoxin was eluted with pure methanol and quantified by HPLC with a reversed-phase C18 column and a fluorimetric spectrum detector. The limit of quantification (LOQ) was 0.1 mg/kg.

#### 2.2.5. Pesticides Residues

Dispersive SPE—Modular QuEChERS (Quick Easy Cheap Effective Rugged Safe) method was used for acetonitrile extraction and purification of pesticide residues CS according to European standards EN 15662:2018 [24]. The compound investigated are listed in Supplementary Table S1. Pesticide residues were analyzed in duplicate by a multimethod procedure using GC-MS/MS and LC-MS/MS with a LOQ (limit of quantification) of 0.01 mg/kg.

### 2.3. Microbiological Analysis

Total Mesophilic Aerobic Bacteria (TMAB), Lactic Acid Bacteria (LAB), Enterobacteriaceae, yeasts and molds and Salmonellae were detected to evaluate the microbiological contamination of coffee silverskin. CS was serially diluted in Quarter-Strength Ringer’s solution (1:10 *w*/*v*) (Basingstoke, UK). An inoculum of 0.1 and 1 mL was then transferred from dilutions into (pour plate method) and onto (spread plate method) appropriate medium. TMAB were counted on Plate Count Agar (PCA) after 48 h of incubation at 30 °C. LABs were counted on deMan Rogosa and Sharp (MRS) Agar after 48 h of incubation at 30 °C. Enterobacteriaceae were counted on Violet Red Bile Glucose Agar (VRBGA) after 48 h of incubation at 37 °C. Yeasts and Molds were counted on Dichloran Rose Bengal Chloramphenicol (DRBC) Agar after 48 h of incubation at 28 °C. Salmonellae were detected according to [25], which involves an initial pre-enrichment step in Buffered Peptone Water at a ratio of 1:10. After incubation at 34–38 °C for 18 h, 0.1 mL was transferred into 10 mL of Rappaport-Vassiliadiscon Soy Broth (RVS) and reincubated at 41.5 °C for 24 h, while 1 mL was used to inoculate 10 mL of Muller-Kauffmann to Tetrationate-novobiocin broth (MKTTn) reincubated subsequently at 37 °C for 24 h. For the next isolation step, Xylose Lysine Deoxycholate (XLD) agar and Brilliance Salmonella Agar Base (BSA) agar plates were seeded by streaking from RVS and MKTTn cultures for each culture broth, which was incubated at 37 °C for 24 h.

### 2.4. Risk Assessment

The risk due to potential consumption of silverskin was assessed through hazard-quotient (HQ) and lifetime cancer risk (LTCR).

HQ based on estimated daily intake (EDI) was calculated through Equations (1) and (2). An HQ > 1, indicates a likely high risk as far as non-carcinogenic adverse effects are concerned.

(1) $$EDI_{k}=\frac{C_{k}\;x\;IR}{BW\;}$$

(2) $$HQ_{k}=\frac{EDI_{k}\;}{TDI_{k}}$$

*C_k_*: Concentration of contaminant k detected in silverskin (mg/kg).

*IR*: Intake Rate of silverskin (kg/day).

*BW*: Body Weight of 70 kg.

*TDI*: tolerable daily intake.

The risk derived from cumulative exposure was calculated through the Hazard Index (Equation (3)). HI > 1 indicates a likely high no-carcinogenic risk due to exposure to multiple contaminants.

(3) $$HQ_{k}=\frac{EDI_{k}\;}{TDI_{k}}$$

LTCR was estimated for carcinogenic contaminants based on their slope factor (SF), as shown in Equation (4). Values above 1 × 10^−4^ are considered unacceptable for the risk of developing cancer over a human lifetime. Whereas values between 1 × 10^−6^ and 1 × 10^−4^ are considered an acceptable range for risk according to [26].

LTCR = (EDI × EF × TE × SF)/AT(4)

EF: Exposure Frequency to the contaminant (350 day/year).

TE: Total Exposure (70 year).

AT: Average Lifetime time for non-carcinogenic risk (TE × 365 day/year).

SF: Slope Factor (mg/kgbw/day)**^−^**^1^ related to each PAE (Table 1).

## 3. Results and Discussion

### 3.1. Chemical Contaminants

During coffee roasting, the high temperature produces a chemical change in coffee beans composition and the formation of new organic compounds as a result of the Maillard reaction and pyrolysis of nonvolatile compounds [6,27]. These include toxic compounds such as polycyclic aromatic hydrocarbons (PAHs) and acrylamide (AA) [28,29]. Our preliminary study [30] evaluated the concentration of process contaminants such as acrylamide (AA), furan, methyl furan and ochratoxin A (OTA) and some heavy metals and PAHs in CS for its multipurpose recycling applications. This study performed an extensive semiquantitative elemental screening of the CS chemical profile. Several classes of contaminants, such as heavy metals, PAHs, process contaminants, OTA, and pesticides, were investigated in *Coffea arabica*, *robusta*, and mixed silverskin. The summary data are listed in Table 1, Table 2, Table 3, Table 4 and Table 5. The contents of the elements analyzed are mostly comparable to each other in the three respective CS samples, except for barium (Ba), copper (Cu), iron (Fe), manganese (Mn) and nickel (Ni). In detail, *robusta* CS has a higher Fe content (442 mg/kg) than *arabica* CS (179 mg/kg), while mixed CS has an intermediate value (319 mg/kg). A similar trend occurs for Cu content, with *robusta* CS (132 mg/kg), mixed CS (106 mg/kg), and *arabica* CS (35.5 mg/kg). In contrast, *arabica* CS shows higher values for Ba and Mn, 60.6 mg/kg and 53.1 mg/kg, respectively. The mixed CS reports for almost all elements values intermediate to the two varieties, except for Ni (2.41 mg/kg), Co (0.68 mg/kg) and V (0.76 mg/kg) with higher values and As (0.21 mg/kg) and Be (0.05 mg/kg) with lower values than the two coffee varieties.

From literature, [10,31,32] assessed the levels of some mineral elements in an unspecified mixture of *arabica* and *robusta* coffee silverskin. Comparing these studies with our mixed CS results, [31] reported higher values for iron (843.30 mg/kg), zinc (22.30 mg/kg), cobalt (21.39 mg/kg), and chromium (1.59 mg/kg), lower value for copper (63.30 mg/kg) while elements such as barium, boron, lead, selenium, cadmium, and tin were in line with ours. Instead, [32] reported higher values for iron (660 mg/kg), zinc (27 mg/kg) and chromium (1.8 mg/kg) and lower values for copper (30 mg/kg). Another study [7] reports higher values of chromium (5.55 mg/kg) while lower values of iron (212 mg/kg) and copper (72.15 mg/kg). Furthermore, from the analyses conducted for our previous study, the arsenic and copper content in the mixed CS was higher, while zinc and iron had lower values than in the current study [30]. These differences are probably due to the different compositions of the coffee blends produced in the roasting companies, which result in a different chemical profile of the silverskin obtained. For example, according to the study [33], a CS pellet consisting probably of 70% *Coffea arabica* and 30% *Coffea canephora*, i.e., the typical coffee blend commercially available in Germany, has a different chemical profile from our mixed CS and the other previously cited works. Once again, the study [33] characterized *arabica* CS with higher values for many elements analyzed, particularly for manganese (145 mg/kg), barium (130 mg/kg) and copper (98 mg/kg) than our *arabica* CS, and *robusta* CS with higher values for barium (73 mg/kg), copper (185 mg/kg), nickel (2.3 mg/kg) and chromium (2.9 mg/kg) than our *robusta* CS. Another study [34], analyzed CS from roasting Coffea arabica green beans and reported higher values for iron (238 mg/kg) and zinc (31.9 mg/kg), while lower values are reported for manganese (46.7 mg/kg). As regards PAHs, their occurrence in coffee silverskin is critical information for risk assessment purposes. They are dangerous ubiquitous organic pollutants, and some are classified as probable human carcinogens due to their toxic, carcinogenic and mutagenic nature [35]. Among the three types of CS analyzed, only *arabica* CS reported the occurrence of Phenanthrene (0.07 mg/kg), Fluoranthene (0.18 mg/kg), and Chrysene (0.06 mg/kg). However, from our results, most of the elements and PAHs in *arabica*, *robusta* and mixed silverskin samples were reported at concentrations <LOQ. Similarly, AA, furan, methyl-furan, and OTA were not detected consistent with our previous investigation [30].

Lastly, analyses were performed to determine the occurrence of pesticides. A study [36] evaluated the life cycle assessment (LCA) of green coffee production in Brazil; the results showed that, in addition to a considerable amount of water, 900 kg of total fertilizer and 10 kg of pesticides are used to produce 1,000 kg of coffee. This information, related to the botanical differences between the two coffee varieties, could influence the presence of pesticides in coffee cherries. Indeed, the depth of the root system is different: the *Coffea arabica* roots penetrate deeper into the soil, while the *robusta* roots are highly concentrated near the soil surface [37]. However, analysis of the multiple pesticides searched (Supplementary Table S1) revealed no outliers, and all values detected were at <LOQ concentrations.

### 3.2. Microbiological Contaminants

For a more comprehensive characterization of CS, the occurrence of some biological contaminants such as Enterobacteriaceae was assessed. These bacteria are biological markers of food safety that could indicate the occurrence of pathogens. A higher Enterobacteriaceae microbial load (0.78 ± 0.16 Log/UFC) was reported in mixed CS. However, all samples reported no occurrence of Salmonella spp. Furthermore, yeasts and molds were isolated from mixed CS, reporting a load of 2 ± 0.21 Log/UFC. Instead, the TMBC was 5.45 ± 0.17 Log/UFC in mixed CS, whereas *robusta* and *arabica* CS reported values of 3.61 ± 0.18 Log/UFC and 3.73 ± 0.22 Log/UFC, respectively. However, it is plausible that the high temperature of the roasting process and the low moisture content of CS (4–7%) [38] limits its microbial load and extends its shelf life [10]. In addition, to date, some studies have evaluated a potential application of CS in the formulation of food products that could ensure healthiness after the baking process. In particular, [39,40] evaluated its applicability in bakery products, whereas an interesting study [15] considered the formulation of a chicken meat burger with CS that would increase the shelf life of the meat due to its antioxidant activities in addition to a fiber supplement and an excellent source of minerals such as calcium, potassium and others.

### 3.3. Risk Assessment

CS is a high-nutritional value product with a fiber content of about 30–70% [41,42,43]. Hence, a content of 5–10 g could provide more than 3 g of fiber allowing the use of the nutritional claims of “source of fiber” in CS-based products according to Regulation (EC) No 1924/2006 [44]. Based on this consideration, a consumption of 5 g and 10 g as food ingredients was supposed for the risk assessment. The EDI of each contaminant was calculated and compared with the corresponding threshold value expressed as TDI (Supplementary Table S2). The EDI was not calculated for compounds with concentrations <LOQ. The ratio between EDI and TDI, known as HQ, was listed in Supplementary Table S2. The HQ ranged from 1.42 × 10^−4^ to 6.92 × 10^−2^, from 1.15 × 10^−4^ to 5.45 × 10^−2^, and from 6.12 × 10^−5^ to 6.50 × 10^−2^ for the consumption of 5 g of *robusta*, mixed, and *arabica* CS, respectively. Instead, the HQ ranged from 2.83 × 10^−4^ to 1.38 × 10^−1^, from 2.29 × 10^−4^ To 1.09 × 10^−1^, and from 1.22 × 10^−4^ to 1.30 × 10^−1^ for the consumption of 10 g of *robusta*, mixed, and *arabica* CS, respectively. Since, in all scenarios, the HQs were below 1, non-carcinogenic risk by exposure to a single contaminant could not be attributed to consumption of silverskin to considered levels. In order of magnitude the values in *robusta* CS were: As > Cu > V > Fe > Hg > B > Mn > Ba > Pb > Ni > Zn > Be > Co > Cr. Similarly, the order in mixed CS was V > As > Cu > Fe > Hg > Ba > B > Ni > Mn > Zn > Be > Co > Cr, whereas *arabica* CS reported As > Cd > Mn > Hg > Ba > Cu > Fe > B > Ni> chrysene > Zn > Be > Co > phenanthrene > fluoranthene > Cr.

**Figure 1:**
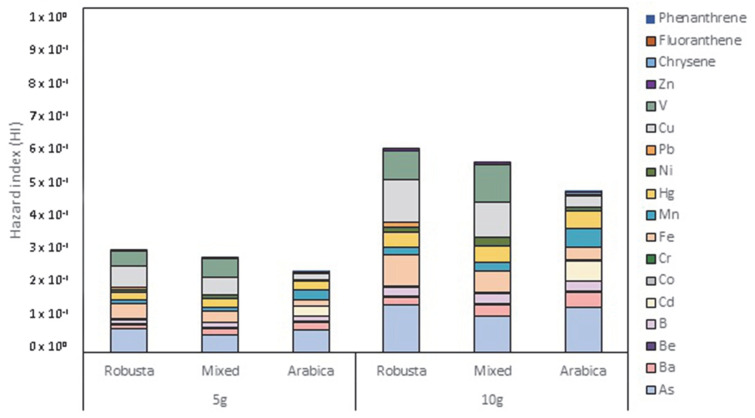
Hazard index (HI) evaluation based on the consumption of 5 g and 10 g of *robusta*, mixed and arabica silverskin.

The HI based on consumption of 5 g and 10 g of CS reported values from 2.96 × 10^−1^ to 5.93 × 10^−1^, from 2.76 × 10^−1^ to 5.53 × 10^−1^, and from 2.34 × 10^−1^ to 4.67 × 10^−1^ for *robusta*, mixed, and *arabica* CS, respectively (Figure 1). The two CS varieties and the mixed CS sample showed values of single and cumulative risk simulation <1, indicating a low probability of non-carcinogenic adverse effects.

Concerning the carcinogenic risk, the estimation of LTCR was only based on As and chrysene since most carcinogenic contaminants were <LOQ. However, the contribution of chrysene was negligible. Instead, As showed a LTCR for the consumption of 5 g and 10 g for *robusta* CS ranged between 2.98 × 10^−5^ and 5.97 × 10^−5^, whereas lower values were reported for mixed CS (2.21 × 10^−5^ and 4.41 × 10^−5^) and *arabica* CS (2.81 × 10^−5^ and 5.61 × 10^−5^). Considering that a carcinogenic risk could occur to values above 1 × 10^−4^ according to USEPA [25], the three types of CS showed a low risk to considered dose as an ingredient.

This risk simulation is based on a conservative approach since 100% bioaccessibility and bioavailability were considered, likely overestimating the risk. Indeed, fiber that is highly present in the silverskin may reduce the bioaccessibility of some elements [45,46,47]. Furthermore, the slope factors of As refer to inorganic form, although foods may also contain the organic one that is less toxic.

## 4. Conclusions

An extensive characterization of essential and toxic elements, REE, pesticides, polycyclic aromatic hydrocarbons, and microorganisms on the two main varieties of CS and their blend was provided. In *robusta* and mixed CS pesticides, REE, process contaminants, OTA, and PAHs were not detected. Similar data were reported for the *arabica* CS sample, although chrysene, phenanthrene, and fluoranthene were detected at low concentrations. Essential and toxic elements showed variability among CS varieties and blended, although As was usually the most representative in all samples. Concerning microorganisms, *arabica* and *robusta* CS reported lower contamination than mixed CS, although Salmonella spp. did not occur in any samples. The handling and collection of the coffee silverskin probably denote slight environmental contamination between the mixed CS obtained from coffee companies and that of *arabica* and *robusta* CS obtained in the laboratory. It should be kept in mind that it is a waste material for companies, and, to date, there are no regulations for its proper sorting and collection. However, the high temperatures of the roasting process allow a reduction of CS microbial load. The risk assessment liked the potential consumption of CS as a food ingredient (5 g and 10 g) and pointed out a low probability of non-carcinogenic and carcinogenic effects. *Robusta* CS showed higher values (risk) for HI and LTCR. As noted by comparison with studies in the literature, differences in the chemical profile of CS can be due to coffee variety, production sites, climatic factors, field treatments, coffee processing methods, and, finally, silverskin storage and collection methods. Overall, this study provides the first evidence of the safety of CS based on determinist assessment. However, future studies should assess the bioaccessibility of elements through in vitro or in vivo digestion to provide more data on the healthiness of CS and its potential application as a novel food.

## Figures and Tables

**Table 1:** Concentrations (mg/kg) of essential, toxic, and rare earth elements in three classes of samples (*n* = 10) of coffee silverskin (CS). Results are expressed as mean ± standard deviation.

Elements	*Robusta* (mg/kg)	Mixed (mg/kg)	*Arabica* (mg/kg)
Antimony Sb	<LOQ	<LOQ	<LOQ
Arsenic As	0.29	0.21	0.27
Barium Ba	28.2	49.4	60.6
Beryllium Be	0.06	0.05	0.06
Boron B	31.3	32.4	33.1
Cadmium Cd	<LOQ	<LOQ	0.15
Cobalt Co	0.45	0.68	0.36
Chromium Cr	0.59	0.48	0.26
Iron Fe	442	319	179
Manganese Mn	20.8	24.6	53.1
Mercury Hg	0.06	0.06	0.07
Nickel Ni	1.08	2.41	0.91
Lead Pb	0.36	<LOQ	<LOQ
Copper Cu	132	106	35.5
Selenium Se	<LOQ	<LOQ	<LOQ
Tin Sn	<LOQ	<LOQ	<LOQ
Thallium Tl	<LOQ	<LOQ	<LOQ
Vanadium V	0.58	0.76	<LOQ
Zinc Zn	17.9	15.8	11.2
Yttrium Y	<LOQ	<LOQ	<LOQ
Lanthanum La	<LOQ	<LOQ	0.07
Cerium Ce	<LOQ	<LOQ	0.07
Praseodymium Pr	<LOQ	<LOQ	<LOQ
Neodymium Nd	<LOQ	<LOQ	<LOQ
Samarium Sm	<LOQ	<LOQ	<LOQ
Europium Eu	<LOQ	<LOQ	<LOQ
Gadolinium Gd	<LOQ	<LOQ	<LOQ
Terbium Tb	<LOQ	<LOQ	<LOQ
Dysprosium Dy	<LOQ	<LOQ	<LOQ
Holmium Ho	<LOQ	<LOQ	<LOQ
Erbium Er	<LOQ	<LOQ	<LOQ
Thulium Tm	<LOQ	<LOQ	<LOQ
Ytterbium Yb	<LOQ	<LOQ	<LOQ
Lutetium Lu	<LOQ	<LOQ	<LOQ

**Table 2:** Concentrations (mg/kg) of polycyclic aromatic hydrocarbons (PAHs) in three classes of samples (*n* = 10) of coffee silverskin (CS). Results are expressed as mean ± standard deviation.

Polycyclic Aromatic Hydrocarbons	*Robusta* (mg/kg)	Mixed (mg/kg)	*Arabica* (mg/kg)
Naphthalene	<LOQ	<LOQ	<LOQ
Acenaphtilene	<LOQ	<LOQ	<LOQ
Acenaphthene	<LOQ	<LOQ	<LOQ
Fluorene	<LOQ	<LOQ	<LOQ
Anthracene	<LOQ	<LOQ	<LOQ
Phenanthrene	<LOQ	<LOQ	0.07
Fluoranthene	<LOQ	<LOQ	0.18
Pyrene	<LOQ	<LOQ	<LOQ
Chrysene	<LOQ	<LOQ	0.06
Benzo(b)fluoranthene	<LOQ	<LOQ	<LOQ
Benzo(k)fluoranthene	<LOQ	<LOQ	<LOQ
Benzo(a)pyrene	<LOQ	<LOQ	<LOQ
Indeno[1,2,3-cd]pyrene	<LOQ	<LOQ	<LOQ
Dibenz[a,h]anthracene	<LOQ	<LOQ	<LOQ
Benzo[ghi]perylene	<LOQ	<LOQ	<LOQ

**Table 3:** Concentrations (mg/kg) of process contaminants in three classes of samples (*n* = 10) of coffee silverskin (CS).

*Process Contaminants*	*Robusta* (mg/kg)	Mixed (mg/kg)	*Arabica* (mg/kg)
Acrylamide (AA)	<LOQ	<LOQ	<LOQ
Furan	<LOQ	<LOQ	<LOQ
Methyl-furan	<LOQ	<LOQ	<LOQ

**Table 4:** Concentrations (mg/kg) of *mycotoxins* in three classes of samples (*n* = 10) of coffee silverskin (CS).

*Mycotoxins*	*Robusta* (mg/kg)	Mixed (mg/kg)	*Arabica* (mg/kg)
Ochratoxin A (OTA)	<LOQ	<LOQ	<LOQ

**Table 5:** Concentrations (mg/kg) of pesticides (showed in Supplementary Table S1) in three classes of samples (*n* = 10) of coffee silverskin (CS).

Pesticides	*Robusta* (mg/kg)	Mixed (mg/kg)	*Arabica* (mg/kg)
**Supplementary Table S1**	<0.01	<0.01	<0.01

## Data Availability

Data will be made available upon request.

## Supplementary Materials

The following supporting information can be downloaded at: https://www.mdpi.com/article/10.3390/foods11182834/s1, Table S1: Multiresidual list of pesticides assessed in silverskin (CS) by GC MS/MS (list 1) and LC MS/MS (list 2); Table S2: Hazard quotient (HQ) based on consumption of 5 g and 10 g of three class samples of coffee silverskin (CS) and the threshold values for each contaminant expressed as tolerable daily intake (TDI, mg/kgbw/day). If not available, TDI was derived from tolerable week intake ^a^ (TWI, mg/kgbw/week), provisional tolerable weekly intake ^b^ (PTWI, mg/kgbw/week), or reference dose ^c^ (RfD, mg/kgbw/day) [48,49,50,51,52,53,54,55,56,57,58,59].
