# Supplementary material for: Coffee Silverskin: Chemical and Biological Risk Assessment and Health Profile for Its Potential Use in Functional Foods
Source: Foods. 2022 Sep 13;11(18):2834. doi: 10.3390/foods11182834 (PMC9498437; doi:10.3390/foods11182834)
Supplement: Supplementary file 1 [file foods-11-02834-s001.zip › foods-1854144-supplementary-PUBLISH.pdf]

Table S1. Multiresidual list of pesticides assessed in silverskin (CS) by GC MS/MS (list 1) and LC MS/MS (list 2)

LIST 1

Acephate	Aclonifen	Acrinathrin	Alachlor
Ametryn	Amitraz	Atrazine	Azinphos ethyl
Azinphos methyl	Azoxystrobin	Benalaxyl	Benfluralin
Benfuracarb	Bifenthrin	Bitertanol	Boscalid
Bromopropylate	Bromuconazole (sum of	Bupirimate	Buprofezin
Cadusafos	diastereoisomer)	Captan	Carbaryl
Carbofuran	Captafol	Carbosulfan	Chiorfenapyr
Chiorfevinphos	Carbophenothion	Chlorothalonil	Chlorpropham
Chiorpyriphos ethyl	Chlormefos	Chlorthal dimetil	Chlozolinate
Chlordane	Chilorpyriphos methyl	Cyproconazole	Cyprodinil
Cyflutrin (sum of isomers)	Cypermethrin (sum of isomers)	Diazinon	Dichlobenil
DDT (sum of p,p' DDT, o,p′	Deltamethrin	Dichlorvos	Diclobutrazol
DDT, p,p′ DDE, and p,p' TDE	Dichloran.	Difenoconazole	Dimethoate (sum of
			Dimethoate
(DDD) expressed as DDT)	Dieldrin (sum of Aldrin	Endosulfan-sulfate (sum of	and Omethoate
Dichiofluania	and Dieldrin expressed	isomer a,b, e sulfate	as Dimethoate)
Dicofol	as Delrin)	expressed as Endosulfan)	Diquat
Diphenylamine	Disulfoton	Ethion	Endrin
Esfenvalerate	Ethiofencarb	Exithiazox	Ethoprophos
Ethoxyguine	Etofenprox	Fenarimol	Famoxadone
Fenamidone	Fenamiphos	Fenhexamid	Fenazaquin
Fenbuconazole	Fenchlorphos	Fenpropidin	Fenitrothion
Fenoxycarb	Fenpropathrin	Fluazifop P.butile	Fenthion
Fenvalerate	Fipronil	Fluvalinate	Flucytrinate
Fludioxonl	Flusilazole	Heptachlor (sum of	Folpet
Furalaxit	Furathiocarb	Heptachlor and Heptachlor	Heptenophos
Hexachlorobenzene	HCH-a	epoxide expressed as	Gamma -
		Heptachlor)	hexachorohexane
			(lindane)
Hexaconazole	haloxyfop methyl	HCH	Iprodione
Iprovalicarb	Imazall	Indoxacarb (sum of Indoxacarb	Lambda-Cyhalothrin
Lenacil	Isophenphos	and its enantiomer R)	Mepanipyrim
Metalaxyl (sum of isomers	Linuron	Kresoxim-methyl	Methiocarb
including Metalaxyl-m)	Methamidophos	Malathion	Metribuzin
Metholachlor	Methomyl	Methidathion	Oxamyl
Myclobutanil	Nuarimol	Methoxiclor	Parathion methyl
Oxyfluorfen	Paclobutrazolo	Oxadixyl	Pertane
Penconazole	Pendimetalin	Parathion ethyl	Phosphamidone
Phorate	Phosalone	Permethrin (sum of isomers)	Profenophos
Pirimicarb	Prochloraz	Phosmet	Propham
Propachior	Propamocarb	Procymidone	Pyraciostrobin
Propiconazole	Propoxur	Propargite	Pyridaphenthion
Pyrazofos	Pyrethrins (technical mixture)	Propyzamide	Quinalfos
Pyrifenox	Pyrimethanil	Pyridaben	Simazine
Quinoxifen	Quintozene	Pyriproxyfen	Tebuconazole
Symetryn	Spiromesifen	Quizalofop ethyl	Terbutryn
Tebutenpyrad	Tecnazen	Sulfotep	Tetramethrin
Tetrachlorvinphos	Tetraconazole	Tefluthrin	Triadimefon e
			Triadimenol (sum of
Thionazin	Tolclofos methyl	Tetradifon	Triadimefon and
			Triadimenol)
Triazophos	Trichlorfon	Tolylfluanid	Trifluralin
Vinclozolin	Zoxamide	Trifioxystrobin	

LIST 2

2,4-D (sum of 2,4-D, its	Abamectin	Acephate	Acetamiprid
salts, esters, and conjugates	Ametoctradin	Atrazine	Azadirachtin
expressed as 2,4-D)	Benthiocarb	Bifenazato (sum of	Boscalid
Aldicarb (sum of Aldicarb,	Bromadiolone	bifenazato and	Butoxycarboxim

Aldicarb- sulfone, and
Aldicarb- sulfoxide
expressed as Aldicarb)
Azoxystrobin

Brodifacoum
Buturon
Chlorantraniliprole
Chlothianidin
Cyantraniliprole
Cymoxanil
Diethofencarb
Dimethomorph

Diuron
Epoxiconazole
Famoxadone
Fenhexamid
Fenpyroximate
Flufenoxuron

Flupyradifurone

Fosthiazate
Imazalil
Iprovalicarb
Isopyrazam
Mepanipyrim
Metalaxyl-m Methiocarb
(sum of Methiocarb,
Methiocarb- sulfone,
Methiocarb-sulfoxide
expressed as
Methiocarb)
Metobromuron
Milbemectina A4
Nitenpyran
Oxamyl-oxime
Pirimicarb
Prometrin
Prosulfocarb
Pyriproxyfen
Spinetoram
(Spirotetramat and its
4 metabolites expressed
as Spirotetramat)

Tebufenpyrad
Thiametoxam
Tolclofos methyl
Zoxamide

Carbaryl
Chlorbromuron
Clethodim
Cyazofamid

Cyromazine
Difenoxuron
Diniconazole
Dodine
Etiofencarb
Fenazaquin
Fenoxycarb
Fenuron

Fluometuron
Flutriafol
Furathiocarb
Imazamox
Isocarbophos
Linuron

Mepronil

Metamitron
Metholachlor, S-
Metoxuron
Monolinuron
Oxadiazon
Oxydemethon methyl
Pirimiphos-ethyl
Propamocarb (sum of
Propamocarb e its
salts expressed as
Propamocarb)
Pymetrozine
Rotenone
Spinosad (sum of
Spinosyn-a,
Spinosyn-d expressed
as Spinosad)
Spiroxamine
Teflubenzuron
Thiobencarb
Tridemorph
(Aquatecide) 2-4 Na
diclorophenoxyacetate

Tiguron

bifenazato- diazene
expressed as bifenazato)
Bromuconazole (sum of
Diastereoisomeri)

Carbendazim (sum of
Benomil and Carbendazim
expressed as Carbendazim)
Chlorotoluron
Clofentezine
Cycloxydim
Demeton S-methyl sulphone
Diflubenzuron

Dioxacarb
Ema B1B
Ethofenprox
Fenbuconazole
Fenpropimorph
Fipronil (sum of Fipronil

and Sulfone metabolite

expressed as Fipronil)
Fluopicolide
Fluxapyroxad
Hexaflumuron
Imidacloprid
lsofenphos-methyl
Lufenuron Metaflumizone
Metazachlor
Methomyl (sum of Methomyl
and Thiodicarb expressed
as Methomyl)
Metrafenone
Monuron
Oxadixyl
Penthiopyrad
Pirimiphos-methyl
Propargite
Pyraclostrobin
Simazine
Spirodiclofen
Sulfoxaflor (sum of isomers)
Thiabendazole
Thiocyclam

Triflumuron

Carbofuran (sum of
Carbofuran and 3-
Hydroxycarbofuran
expressed as
Carbofuran)
Chlorsulfuron
Cyanophenfos
Cyflufenamid
Dichlofluanid
Diflufenican
Ditalimfos
Emamectina benzoato
(Emamectina B1A
expressed
as Emamectina)
Etoxazole
Fenbutatin oxide
Fenpyrazamine
Flonicamid (sum of
Flonicamid, TFNA, and
TFNG
exoressed as
Flonicamid)
Fluopyram Formetanate
Hexythiazox
Indoxacarb (sum of
indoxacarb and its
enantiomer R)
lsoproturon
Mandipropamid
Metalaxyl (sum of
isomers including
Metalaxyl-m)
Methamidophos
Methoxyfenozide
Milbemectina A3
Neburon
Oxamyl
Phenmedipham
Promecarb
Propoxur
Pyraflufen-ethyl
Milbemectina (sum of
Milbemectina A4
and Milbemectina A3
expressed as
Milbemectina
Spiromesifen
Tebufenozide
Thiacloprid
Thiophanate methyl
Triforine

Table S2. Hazard quotient (HQ) based on consumption of 5g and 10g of three class samples of coffee silverskin (CS) and the threshold values for each contaminant expressed as tolerable daily intake (TDI, $mg/kg_{bw}/day$). If not available, TDI was derived from tolerable week intake[a] (TWI, $mg/kg_{bw}/week$), provisional tolerable week intake[b] (PTWI, $mg/kg_{bw}/week$), or reference dose[c] (RfD, $mg/kg_{bw}/day$).

	HQ (IR: 5g)			HQ (IR: 10g)			TDI ($mg/kg_{bw}/day$)	Reference
**Contaminant**	***Robusta***	**Mixed**	***Arabica***	***Robusta***	**Mixed**	***Arabica***		
**As**	2.08E-05	5.12E-02	6.50E-02	4.15E-05	1.02E-01	1.30E-01	3.00E-04	[48]
**Ba**	2.02E-03	1.77E-02	2.17E-02	4.03E-03	3.53E-02	4.33E-02	2.00E-01	[49]
**Be**	4.29E-06	1.79E-03	2.14E-03	8.57E-06	3.57E-03	4.29E-03	2.00E-03	[50]
**B**	2.24E-03	1.36E-02	1.39E-02	4.47E-03	2.72E-02	2.78E-02	1.70E-01	[51]
**Cd**	NA	NA	3.00E-02	NA	NA	6.00E-02	2.50E-03[b]	[52]
**Co**	3.21E-05	1.61E-03	8.57E-04	6.41E-05	3.22E-03	1.71E-03	3.00E-02[c]	[53]
**Cr**	4.25E-05	1.15E-04	6.12E-05	8.50E-05	2.29E-04	1.22E-04	3.00E-01	[54]
**Fe**	4.51E-02	3.25E-02	1.82E-02	9.02E-02	6.50E-02	3.65E-02	7.00E-01	[48]
**Mn**	1.48E-03	1.26E-02	2.71E-02	2.97E-03	2.51E-02	5.42E-02	1.40E-01	[48]
**Hg**	4.33E-06	2.34E-02	2.59E-02	8.65E-06	4.67E-02	5.19E-02	1.30E-03[a]	[55]
**Ni**	7.74E-05	1.32E-02	5.02E-03	1.55E-04	2.65E-02	1.00E-02	1.30E-02	[56]
**Pb**	2.57E-05	NA	NA	5.14E-05	NA	NA	2.52E-02[b]	[57]
**Cu**	9.43E-03	5.04E-02	1.69E-02	1.89E-02	1.01E-01	3.38E-02	1.50E-01	[58]
**V**	4.13E-05	5.45E-02	NA	8.26E-05	1.09E-01	NA	1.00E-03	[48]
**Zn**	1.28E-03	3.75E-03	2.66E-03	2.56E-03	7.50E-03	5.33E-03	3.00E-01	[48]
**Chrysene**	NA	NA	2.98E-03	NA	NA	5.95E-03	1.44E-03[c]	[59]
**Fluoranthene**	NA	NA	3.21E-04	NA	NA	6.43E-04	4.00E-02[c]	[48]
**Phenanthrene**	NA	NA	7.62E-04	NA	NA	1.52E-03	6.56E-03[c]	[59]
